# Oxygen and RNA in stress-induced mutation

**DOI:** 10.1007/s00294-017-0801-9

**Published:** 2018-01-02

**Authors:** Raul Correa, Philip C. Thornton, Susan M. Rosenberg, P. J. Hastings

**Affiliations:** 1 Department of Molecular and Human Genetics, Baylor College of Medicine, 1 Baylor Plaza, Houston, TX 77303 USA; 2 Dan L. Duncan Comprehensive Cancer Center, Baylor College of Medicine, 1 Baylor Plaza, Houston, TX 77303 USA; 3 Department of Biochemistry and Molecular Biology, Baylor College of Medicine, 1 Baylor Plaza, Houston, TX 77303 USA; 4 Department of Molecular Virology and Microbiology, Baylor College of Medicine, 1 Baylor Plaza, Houston, TX 77303 USA

**Keywords:** Mutagenic break repair, Mutation, R-loop, Break-induced replication, Stress-response, Reactive oxygen species

## Abstract

Mechanisms of mutation upregulated by stress responses have been described in several organisms from bacteria to human. These mechanisms might accelerate genetic change specifically when cells are maladapted to their environment. Stress-induced mutation mechanisms differ in their genetic requirements from mutation in growing cells, occurring by different mechanisms in different assay systems, but having in common a requirement for the induction of stress-responses. Here, we review progress in two areas relevant to stress-response-dependent mutagenic DNA break repair mechanisms in *Escherichia coli*. First, we review evidence that relates mutation to transcription. This connection might allow mutagenesis in transcribed regions, including those relevant to any stress being experienced, opening the possibility that mutations could be targeted to regions where mutation might be advantageous under conditions of a specific stress. We review the mechanisms by which replication initiated by transcription can lead to mutation. Second, we review recent findings that, although stress-induced mutation does not require exogenous DNA-damaging agents, it does require the presence of damaged bases in DNA. For starved *E. coli*, endogenous oxygen radicals cause these altered bases. We postulate that damaged bases stall the replisome, which, we suggest, is required for DNA-polymerase exchange, allowing the action of low-fidelity DNA polymerases that promote mutation.

## Introduction

Stress-induced mutagenesis encompasses the mechanisms of transient genomic instability up-regulated by stress-responses. Stress-response regulation suggests that these mechanisms accelerate evolution in organisms that are ill adapted to their environment, that is, that are stressed. Stress-induced mutation mechanisms are found throughout biology, varying, in detail, between organisms but having in common the requirement for activation of one or more stress-responses which promotes transient hypermutation [reviewed by (Fitzgerald et al. 2017)].

Mutagenic break repair (MBR) includes two molecular mechanisms of stress-induced mutation in *Escherichia coli*, both of which have been elaborated in detail [reviewed by (Fitzgerald et al. 2017)]. A compendium of work on stress-induced mutation demonstrates detailed mechanisms that upregulate mutation transiently, causing bursts of genome instability, a departure from early neo-Darwinian assumptions (Mayr 1985) of constant, gradual mutation rates, and evolution [reviewed by (Fitzgerald et al. 2017)]. Of particular importance is the finding of similar mechanisms in other assays and other organisms (Fitzgerald et al. 2017; Castro-Cerritos et al. 2017), most notably the chromosomal Tet assay in *E. coli* discussed below. Here, we review and discuss two aspects of stress-induced MBR mechanisms in *E. coli*:, their dependence on DNA breaks stimulated by transcription, and thus the possibility that mutation is targeted to genomic regions where it is needed, and the finding that spontaneous mutation in non-growing cells happens only when there are damaged bases in the DNA.

## Assays for stress-inducible mutagenic break repair

In the Lac assay (Cairns and Foster 1991), reversion of a *lac* + 1 basepair frameshift mutation happens over days of starvation, regulated by at least three stress responses. The reversions are of two types: − 1 basepair indels (“point mutations”) that restore reading frame, and amplification of the leaky *lac* allele to multiple copies (Hastings et al. 2000). Thus, the Lac assay monitors two of the major processes of genome evolution: change in DNA sequence and change in chromosome structure. Transposon movement, which occurs in response to environmental stimuli in *E. coli* (Schnetz et al. 1987; Hall 1998; Haniford 2006; Kim et al. 2016), is not monitored in the mutation assays discussed here.

The point mutagenesis (base-substitution and indel) mechanism requires DNA double-strand breaks (DSBs) and the enzymes of DSB repair by homologous recombination (Harris et al. 1994, 1996; Foster et al. 1996; Ponder et al. 2005) It also requires low-fidelity DNA polymerases and the activity of stress-response regulators, the RpoS (σ^S^) general stress-response (Layton and Foster 2003; Lombardo et al. 2004), and the SOS DNA damage response (McKenzie et al. 2000) [reviewed by (Fitzgerald et al. 2017)]. Mutation at *lac* also requires the RpoE (σ^E^) unfolded protein response, apparently for the generation of some spontaneous DSBs (Gibson et al. 2010).

Chromosomal rearrangement, measured by amplification of the *lac* region, is seen as a tandem array of sequences joined by microhomology of about 3–15 base pairs, too short for homologous recombination (Hastings et al. 2000, 2004; Slack et al. 2006). Amplification is postulated to involve the initial duplication formation by non-homologous recombination, followed by expansion to multiple copies by unequal crossing-over (Slack et al. 2006). Amplification requires DSBs (Ponder et al. 2005; Slack et al. 2006; Wimberly et al. 2013) and most of the same proteins as point mutagenesis (Slack et al. 2006), except that it does not require the SOS DNA-damage response or the SOS-upregulated DNA polymerase IV (McKenzie et al. 2001); but, unlike point mutation, amplification requires DNA polymerase I (Slack et al. 2006). Both point mutagenesis and amplification are mechanisms of mutagenic break repair (MBR) (Rosenberg et al. 2012; Rogers et al. 2015; Fitzgerald et al. 2017).

In the Lac assay, starvation on lactose is both the stressor and selects the Lac^+^ mutant readout, making it possible that selection is part of the mechanism. This potential ambiguity was overcome by use of an alternative assay that measures reversion during starvation of a frameshift mutation in a *tetA* gene required for tetracycline resistance (Ponder et al. 2005; Shee et al. 2011). In the Tet assay, cells are starved, rescued from starvation, and only then are exposed to tetracycline, so that selection for resistance has no role in the mutation mechanism. Mutation in the Tet assay also requires DSBs, which are provided by a site-specific endonuclease (I-SceI), DSB repair by the enzymes of homologous recombination, the RpoS and SOS stress response regulators, and up-regulation of error-prone DNA polymerases (Ponder et al. 2005; Shee et al. 2011). In addition, MBR measured in the Tet assay, and in a chromosomal assay for base substitutions, accounts for about half of spontaneous mutation in starved cells with no I-SceI endonuclease, in which it results from spontaneous DSBs (Shee et al. 2011).

The Tet assay is used either with the *tetA* gene in an F’ plasmid (Ponder et al. 2005) or in the *E. coli* chromosome in plasmid-free cells (Shee et al. 2011), both of which report stress-response dependent MBR. The generality of stress-induced mutation is also demonstrated by findings of similar mechanisms in other organisms, including human cancer cells [reviewed by (Fitzgerald et al. 2017)].

## Is stress-induced mutation targeted?

Although early studies raised the possibility that what we now know to be stress-induced MBR might occur only in genes the functions of which were selected [discussed by (Stahl 1988, 1990)], this idea was retired by the demonstration that many unselected mutations occur throughout the genome in a sub-population of starved cells (Torkelson et al. 1997; Gonzalez et al. 2008). However, it might have been premature to conclude that mutations fall irrespective of where they are needed. First, there is evidence that not all Lac^+^ revertants belong to the hypermutating sub-population (Rosche and Foster 1999). Second, evidence is emerging of transcriptional promotion of MBR, and one would suppose that genes required to counter a stress would be preferentially transcribed under conditions of that stress. This was first described in assays that measured reversion to prototrophy that appeared to target genes for amino-acid synthesis during starvation for the amino acids (Hall 1990; Reimers et al. 2004). Third, the many mechanisms by which transcription affects mutation in other conditions, assays and organisms has been reviewed (Jinks-Robertson and Bhagwat 2014). Fourth, transcriptional R-loops are precursors to DSBs in MBR in the Lac assay (Wimberly et al. 2013).

**Fig. 1:**
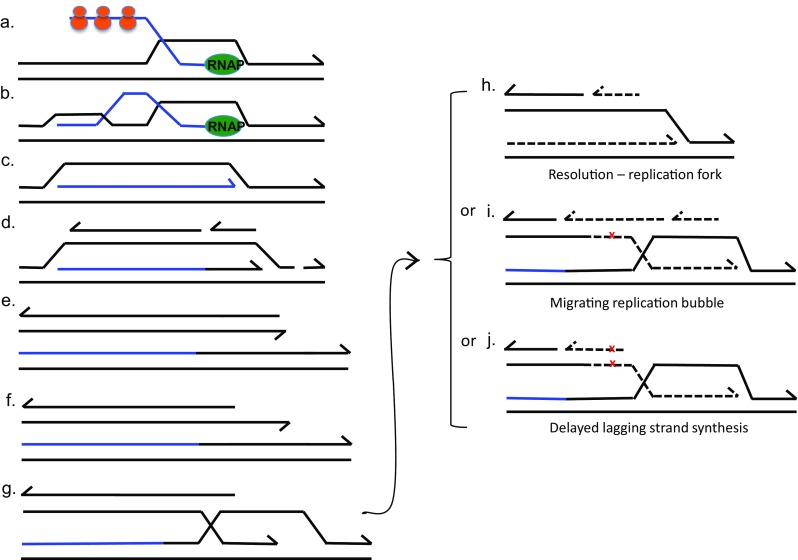
A proposed mechanism of mutagenesis targeted to transcribed regions. The possible mechanism by which transcription leads to BIR was described by (Wimberly et al. 2013). **a** Normally, ribosomes (orange balls) prevent the transcript (blue line) from being incorporated into supercoiled DNA (black line) behind the transcription complex. **b** When the transcript is not protected by ribosomes the transcript can be taken up by the supercoiled DNA behind the transcription complex (Masse and Drolet 1999). **c** Mfd protein removes the RNA polymerase (RNAP) (Park et al. 2002), allowing a complete R-loop to form. **d** The R-loop primes a unidirectional replication fork (Kogoma 1997). **e** When the replication fork encounters a single-strand nick in a template strand, the DNA arm breaks off the fork (Kuzminov 1995, 2001). **f** Replication is restarted by break-induced replication (BIR) by 5′ resection at the broken end and, **g** invasion of the sister molecule by the 3′ end (Hastings et al. 2009b), initiating a replication fork that is subject to high frequency point mutation (Deem et al. 2011), and template switching (Smith et al. 2007). BIR might proceed in three different ways, with evidence for each of them: **h** The Holliday junction might be cleaved by resolvase so that a normal non-mutagenic replication fork is formed and mutation is limited (Mayle et al. 2015), or **i** the replication bubble migrates with both leading and lagging strand synthesis pulling a Holliday junction (Xia et al. 2016), resulting in conservative distribution of old and new DNA strands (Motamedi et al. 1999) with loss of the direction of mismatch repair by old DNA strands, giving increased mutation (Kuzminov 1995), or **j** the D-loop migrates with lagging-strand synthesis delayed so that the nascent leading strand becomes the lagging-strand template, resulting in conservative segregation of strands and high mutation rate because there are no mismatches and so no opportunity for mismatch repair (Malkova and Ira 2013; Saini et al. 2013). The red x represents a DNA polymerase error. Dashed lines represent BIR DNA synthesis. RNAP: RNA polymerase. Half arrows indicate 3′ ends. Orange bodies represent ribosomes

An R-loop is a three-stranded structure in which RNA displaces one strand of DNA, base-pairing with its complement. R-loops can be formed by incorporation of a transcript into DNA behind the RNA polymerase (RNAP, Fig. 1a–c). R-loops can initiate DNA synthesis without the involvement of a replication origin (Kogoma 1997) (Fig. 1d). R-loops in an F’ plasmid are proposed to form an adventitious replication fork that encounters a single-stranded DNA nick formed by the plasmid transfer endonuclease (Wimberly et al. 2013) (Fig. 1d, e). When a replication fork encounters a nick, the fork breaks (Kuzminov 2001) producing one DSB end (Fig. 1e), and this might provide a source of the DSBs required for MBR in the Lac assay (Wimberly et al. 2013).

Repair of DSBs in the *E. coli* chromosome focuses mutations to the vicinity of the DSB, most mutation falling within about five kilobase pairs of the break, declining exponentially to about 60 kb from the break on either side (Shee et al. 2012). Hence, we postulate that there might be a pathway whereby attempted transcription under starvation conditions leads to abortion of transcription and R-loop formation. This, in turn, leads to DSB formation via adventitious R-loop-mediated replication (Kogoma 1997) encountering a nicked template (Kuzminov 2001). Thus, repair of the broken replication fork by break-induced replication (BIR) can lead to mutation in the vicinity of the aborted transcription, and hence in a region where transcription was induced under conditions of stress (Wimberly et al. 2013). This mechanism is illustrated in Fig. 1, based on mechanisms described in (Wimberly et al. 2013).

## Mechanisms of BIR mutagenesis

BIR is a highly mutagenic mechanism in yeast (Smith et al. 2007; Deem et al. 2011). It is of interest that a replication fork proposed to be initiated by an R-loop appears not to have the mutagenic properties associated with BIR, in that an R-loop (and so replication) is not sufficient for mutagenesis; mutagenesis requires the R-loop plus a nick that becomes a DSB, which is then repaired (Wimberly et al. 2013). Perhaps the difference between replication and BIR is that BIR involves D-loop formation, which can lead to conservative DNA segregation of old and new DNA strands (Motamedi et al. 1999; Donnianni and Symington 2013; Malkova and Ira 2013; Saini et al. 2013; Wilson et al. 2013; Roumelioti et al. 2016) (Fig. 1i,j).

BIR in yeast initially involves the use of alternative polymerases (Deem et al. 2011) prone to making replication errors. Many of these errors are presumably removed by mismatch repair. However, BIR structures might limit the efficiency of mismatch repair. BIR could result in three different outcomes as illustrated in Fig. 1h–j. First, the Holliday junction shown in Fig. 1g might be cleaved by a resolvase, allowing formation of a conventional replication fork, with semiconservative distribution of old and new DNA strands that might not generate hypermutation (Fig. 1h). The evidence for this is that the yeast resolvase Mus81 limits the length of mutation resulting from BIR during mitosis (Mayle et al. 2015). The length of mutagenesis is also limited by an oncoming replication fork (Mayle et al. 2015). Second, the D-loop might migrate with the replication fork as a replication bubble that pulls a Holliday junction (Motamedi et al. 1999; Hastings et al. 2009a; Saini et al. 2013; Xia et al. 2016) as shown in Fig. 1i. This migration proceeds possibly to the telomere in yeast (Saini et al. 2013) and is shown to reach the replication terminus in *E. coli*, megabases away from the DSB at which it was initiated (Xia et al. 2016). The mutagenicity of this mode of BIR has been postulated to result in *E. coli* from the loss of direction of mismatch repair by the distinction between old and new DNA that results from conservative distribution of new material behind the migrating D-loop bubble (Kuzminov 1995). However, in *E. coli*, the mutagenesis does not continue from a DSB all the way to the chromosomal replication terminus as this view would predict. Mutation induced by double-strand breaks extends for about 1 megabase from the double-strand cut (Shee et al. 2012), while the Holliday junction thought to be part of a D-loop can be found at the replication terminus more than two megabases from the double-strand cut (Xia et al. 2016). This suggests that BIR might be discontinuous, requiring intermittent restarting that involves the use of alternative DNA polymerases of low fidelity, as BIR initiation does in yeast (Deem et al. 2011).

A third possibility, illustrated in Fig. 1j, is that during BIR, lagging strand replication is delayed until the D-loop has advanced. In yeast, this can leave up to 30 kb of single-stranded DNA (Saini et al. 2013). This might cause the nascent leading strand to become the template for lagging strand synthesis, thus fixing errors made on the leading strand because there is no longer the possibility of mismatch repair (Malkova and Ira 2013). It is also likely that long lengths of single-stranded DNA will be subject to high mutation rates because no complementary strand is present to provide a template for repair of damaged bases (Roberts et al. 2012).

Recently another unexpected example of transcriptional control of genomic change was reported (Hull et al. 2017). It concerns expansion from a few copies of a gene to many copies as is postulated for amplification at the *lac* locus. In yeast, many strains have several tandem copies of the CUP1 locus which, when highly expressed, bestows resistance to high concentrations of copper. This expansion (and contraction) happens by unequal crossing-over, also known as non-allelic homologous recombination (NAHR). The authors show that in the genetic context of *CUP1*, induction of the bidirectional promoter near a known replication fork stalling-site leads to gene copy number alteration (CNA). Further, the transcriptionally induced CNA is dependent on acetylation of histone H3 lysine 56, a mark known to reduce the processivity and synthesis fidelity of restarted replication forks following stalling and collapse. When BIR is initiated in a region in which there are multiple copies of a sequence, the template switching associated with the first few kb of BIR (Smith et al. 2007) might cause synthesis to restart on a copy of the sequence that is not in a position allelic to the copy where the break occurred, thus changing the copy number either up or down. Although these copy-number changes were not shown to involve stress responses, this mechanism adds an exciting extension to our knowledge of how the environment can regulate genomic change in adaptive and, hence, evolutionarily important ways.

## Mutagenic break repair requires base damage in DNA

Translesion synthesis (TLS) DNA polymerases insert nucleotides opposite damaged DNA bases (“lesions”) and/or extend synthesis from primers with lesion:undamaged-base mismatches. They allow DNA replication to proceed while leaving the damaged base(s) in the DNA. Their ability to bypass lesions is achieved by active sites that permit distorted DNA, and by their lack of intrinsic proofreading ability (Kobayashi et al. 2002; Kokoska et al. 2002), which make TLS polymerases inaccurate during replication of undamaged DNA. It is, therefore, not surprising that overproduction of TLS polymerases is highly mutagenic (Kim et al. 1997; Wagner et al. 1999).

Overproduction of TLS polymerases is a critical component of stress-induced MBR in *E. coli*. DNA polymerase IV is up-regulated transcriptionally tenfold by induction of the SOS DNA-damage response (Kim et al. 2001), and twofold by activation of the RpoS general stress response (Layton and Foster 2003), and this over-expression is required for mutagenesis (McKenzie et al. 2001; Galhardo et al. 2009) and explains most of the role of the SOS response in MBR (Galhardo et al. 2009).

Because overproduction of TLS polymerases is mutagenic, overexpression during stress-induced mutagenesis seemed to be a sufficient explanation for the TLS polymerase-dependent mutation in stressed cells. However, while studying the mutational roles of proteins that attach to DNA in *E. coli*, we found correlations between mutagenesis and the presence of reactive oxygen species (ROS) (Moore et al. 2015). Specifically, H-NS, a global gene regulator (repressor), is required for MBR *via* its repression of SodB, an antioxidant enzyme that reduces levels of superoxide. We showed that the requirement for H-NS is completely suppressed by deletion of *sodB* although the *sodB* deletion itself does not have a mutator phenotype (Moore et al. 2015). Thus, H-NS positively regulates ROS level and mutagenesis. Second, Dps, a stationary-phase nucleoid structural protein, which also sequesters iron, hydrogen peroxide and hydroxyl radicals from damaging macromolecules (Zhao et al. 2002; Bellapadrona et al. 2010), is a repressor of MBR (Moore et al. 2015). We showed that the elevated mutation in a *dps* deletion strain is suppressed by constitutive expression of the OxyR regulon (Moore et al. 2017), which encodes alkylhydroperoxidase and a catalase that remove hydrogen peroxide from cells. Thus Dps negatively regulates ROS levels and mutagenesis. Positive and negative regulation of ROS and mutation in parallel suggested that mutation rate is regulated by regulation of ROS, and that stress-response control of ROS levels might be a mechanism by which stress responses regulate mutation.

At present, we know that the SOS response upregulates mutation by its transcriptional upregulation of Pol IV (Galhardo et al. 2009); the σ^E^ membrane stress response regulator promotes MBR by promoting some spontaneous DNA breaks (Gibson et al. 2010); the Hfq RNA chaperone promotes MBR by down-regulating mismatch repair via small RNAs (Kavita et al. 2017); but how the RpoS general stress response promotes mutation is unknown, except that if the RpoS transcriptional activator is supplied in unstressed cells, DNA break repair then becomes mutagenic using Pol IV (Ponder et al. 2005; Shee et al. 2011). This means that stress itself is not needed for mutagenesis, the RpoS response is sufficient without a stressor inducing it.

Following up on these findings we demonstrated that stress-induced mutation in *E. coli* happens only when there are damaged bases in the DNA (Moore et al. 2017). These damaged bases stem predominantly from oxidation by ROS of deoxyguanosine triphosphate in the nucleotide pool. Chemical scavenging of ROS and overexpression of ROS scavenging enzymes both produce large reductions in the levels of stress-induced mutation. ROS do not affect stress-induced mutation *via* protein damage, induction of DNA double-strand breaks, induction of the RpoS and SOS stress responses or SOS error-prone DNA polymerase IV, or saturation of mismatch repair capacity (Moore et al. 2017). Surprisingly, mutation is greatly reduced by overexpression of *mutM*, encoding 8-oxo-guanine (8-oxo-dG) DNA glycosylase that excises 8-oxo-dG from DNA (Michaels et al. 1992; Tajiri et al. 1995). Thus, the 8-oxo-dG has to remain in DNA for mutation to occur. We showed that this promotion of MBR mutagenesis is not specific to oxidatively damaged bases, but can be substituted by lesions induced by ultraviolet irradiation or by the alkylating agent methyl methanesulphonate. Thus, MBR requires base lesions in the DNA, not specifically oxidized bases.

**Fig. 2:**
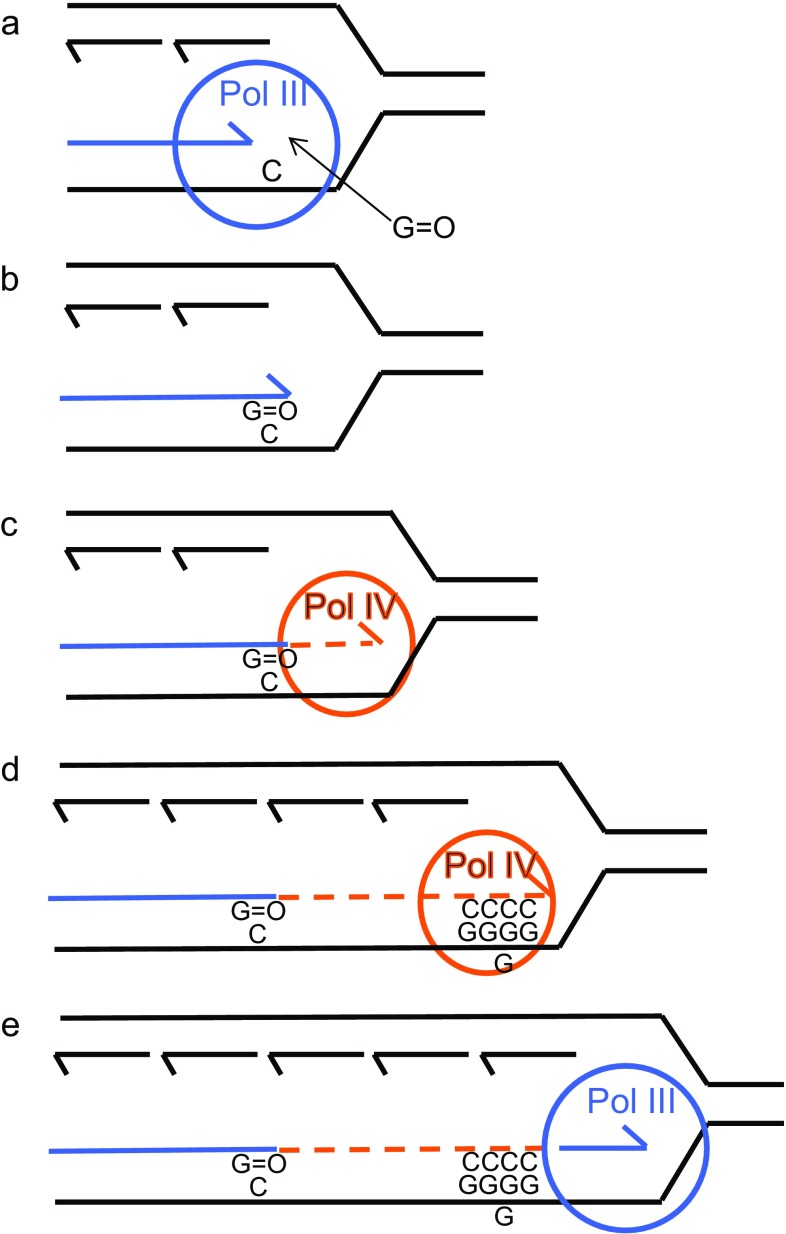
Hypothesis: Damaged bases in DNA might promote mutagenic break repair by allowing DNA polymerase exchange. We show how Pol IV could make − 1 basepair deletions when oxidized guanine is correctly incorporated into DNA opposite template cytosine (Moore et al. 2017). **a** At a repair replisome, the replicative DNA polymerase Pol III incorporates 8-oxo-guanine (G=O) opposite template C (Moore et al. 2017). **b** Pol III does not extend from the 8-oxoG:C base pair efficiently (Yamada et al. 2012), causing the replisome to stall, and Pol III to leave the replisome active site (Markkanen et al. 2012). **c** DNA polymerase Pol IV acquires the replisome active site (Heltzel et al. 2012) and can extend from the 8-oxoG:C base pair (orange). **d** The active site of Pol IV can accommodate extrahelical bases, shown here as an extrahelical G in a run of five Gs (Kobayashi et al. 2002; Kokoska et al. 2002), which results in a − 1 bp deletion because GGGGG is replicated to CCCC. Pol zeta might play a similar role in yeast [reviewed by (Szwajczak et al. 2017)]. **e** Because Pol IV has low processivity, about 400 basepairs (Wagner et al. 2000), it leaves the replisome and Pol III resumes accurate replication (blue). Pol III is shown as a blue circle, Pol IV as an orange circle. Lines represent single DNA strands. Half arrows indicate 3′ DNA ends. Parental DNA is shown as black, new Pol III synthesis in blue and new Pol IV synthesis in orange

We advanced the following hypothesis for the role of DNA damage in MBR (Moore et al. 2017): DNA polymerases in the replisome are attached to the β-clamp (PCNA in eukaryotes). The β-clamp holds more than one DNA polymerase at a time, only one of which is in the active position (Indiani et al. 2005; Fujii and Fuchs 2007; Kath et al. 2014). When the replicative polymerase stalls, another DNA polymerase might, we suggested, come to occupy the active site on the β-clamp, and polymerase exchanges might continue until a processive polymerase is able to resume synthesis. One might expect that the relative abundance of DNA polymerases might influence which is able to win the active site. Thus, if error-prone DNA polymerases are upregulated, they would more often be active, and so there would be more polymerase errors. The finding that base lesions in DNA are required for MBR suggests that abundance is not sufficient to allow an error-prone polymerase to become active. We suggest that it is also necessary to stall the highly processive replicative polymerase, thus allowing DNA polymerase exchange on the β-clamp. An example of how base lesions might promote mutation is illustrated in Fig. 2. We do not know how broadly these ideas might apply, specifically whether base damage in DNA also promotes replication errors in growing cells, though most mutagenesis in growing *E. coli* cells is independent of DNA polymerase IV (McKenzie et al. 2001, 2003), the major mutagenic DNA polymerase of MBR (McKenzie et al. 2001; Ponder et al. 2005; Galhardo et al. 2009; Shee et al. 2011).

ROS are not merely damaging byproducts of oxidative metabolism. ROS also play roles in cell signaling *via* different redox targets, and ROS are required in the immune system and in stress responses that defend hosts from non-oxidative threats (Bogdan et al. 2000; Droge 2002; Valko et al. 2007; Holmstrom and Finkel 2014; Horn et al. 2017). ROS also regulate normal blood cell differentiation during development (Owusu-Ansah and Banerjee 2009). Further, exogenously supplied ROS can act as terminal electron acceptors for anoxically respiring bacteria, allowing their growth on non-fermentable carbon sources (Khademian and Imlay 2017). The apparent role of ROS in accelerated evolution described here might be another example of the co-opting of this metabolic byproduct for biological purposes. Conversely, this knowledge of the role of ROS in stress-inducible mutagenesis opens the possibility of inhibiting evolution under conditions where it is disadvantageous to human health, as, for example, in the evolution of pathogen resistance to antibiotics and evasion of the immune system, or cancer progression and cell resistance to chemotherapeutic agents (Rosenberg and Queitsch 2014; Fitzgerald et al. 2017). The challenge now is to discover the source and regulation of ROS, and so to relate the physiology of stressed cells to the mechanisms targeting stress-induced mutagenesis in time, to times of stress, and in genomic space.
